# The *Non-Coding RNA* Journal Club: Highlights on Recent Papers—12

**DOI:** 10.3390/ncrna9020028

**Published:** 2023-04-18

**Authors:** Patrick K. T. Shiu, Mirolyuba Ilieva, Anja Holm, Shizuka Uchida, Johanna K. DiStefano, Agnieszka Bronisz, Ling Yang, Yoh Asahi, Ajay Goel, Liuqing Yang, Ashok Nuthanakanti, Alexander Serganov, Suresh K. Alahari, Chunru Lin, Barbara Pardini, Alessio Naccarati, Jing Jin, Beshoy Armanios, Xiao-bo Zhong, Nikolaos Sideris, Salih Bayraktar, Leandro Castellano, André P. Gerber, He Lin, Simon J. Conn, Doha Magdy Mostafa Sleem, Lisa Timmons

**Affiliations:** 1 Division of Biological Sciences, University of Missouri, Columbia, MO 65211, USA; 2 Center for RNA Medicine, Department of Clinical Medicine, Aalborg University, DK-2450 Copenhagen SV, Denmark; 3 Department of Experimental Clinical Research, Rigshospitalet, DK-2600 Glostrup, Denmark; 4 Metabolic Disease Research Unit, Translational Genomics Research Institute, Phoenix, AZ 85004, USA; 5 Mossakowski Medical Research Institute, Polish Academy of Sciences, 02-106 Warsaw, Poland; 6 Department of Medical Genetics and Molecular Biochemistry, Lewis Katz School of Medicine at Temple University, Philadelphia, PA 19140, USA; 7 Department of Molecular Diagnostics and Experimental Therapeutics, Beckman Research Institute of City of Hope, Biomedical Research Center, Monrovia, CA 91016, USA; 8 City of Hope Comprehensive Cancer Center, Duarte, CA 91010, USA; 9 Department of Molecular and Cellular Oncology, The University of Texas MD Anderson Cancer Center, Houston, TX 77030, USA; 10 Department of Biochemistry and Molecular Pharmacology, New York University Grossman School of Medicine, New York, NY 10016, USA; 11 Department of Biochemistry and Molecular Biology, LSUHSC School of Medicine, New Orleans, LA 70112, USA; 12 Italian Institute for Genomic Medicine (IIGM), c/o IRCCS Candiolo, Candiolo, 10060 Turin, Italy; 13 Candiolo Cancer Institute, FPO-IRCCS, Candiolo, 10060 Turin, Italy; 14 Department of Pharmaceutical Sciences, School of Pharmacy, The University of Connecticut, 69 N Eagleville Road, Storrs, CT 06269, USA; 15 School of Life Sciences, University of Sussex, Falmer, Brighton BN1 9QG, UK; 16 Department of Surgery and Cancer, Imperial Centre for Translational and Experimental Medicine (ICTEM), Imperial College London, London W12 0NN, UK; 17 Department of Microbial Sciences, School of Biosciences and Medicine, Faculty of Health and Medical Sciences, University of Surrey, Guildford GU2 7XH, UK; 18 Flinders Health and Medical Research Institute, College of Medicine & Public Health, Flinders University, Bedford Park, SA 5042, Australia; 19 Department of Molecular Biosciences, The University of Kansas, 1200 Sunnyside Avenue, Lawrence, KS 66045, USA

## 1. Introduction

We are delighted to share with you our twelfth Journal Club and highlight some of the most interesting papers published recently. We hope to keep you up-to-date with non-coding RNA research that extends beyond your study area. The *Non-Coding RNA* Scientific Board wishes you an exciting and fruitful read.

## 2. Upregulation by microRNAs: More Common Than You Think

### Highlight by Patrick K. T. Shiu

microRNAs (miRNAs) are primarily known for their ability to silence gene expression. In a recent issue of *ACS Central Science*, results from Jame-Chenarboo and others challenge the conventional wisdom that upregulation by miRNAs is rare [1].

In this study, the authors identified the miRNAs associated with two human glycosylation enzymes, ST6GAL1 and ST6GAL2. Surprisingly, the majority (76%) of the ST6GAL1 miRNA hits increase protein expression in HEK-293T (actively dividing) cells. For ST6GAL2, upregulatory miRNAs were also discovered, but in a smaller proportion (31%). For several upregulatory miRNAs, mutation of the corresponding binding site in the 3′-untranslated region eliminates the upregulation, confirming that the effect is through direct miRNA–mRNA interaction. Finally, AGO2 and FXR1 were found to be essential for the observed upregulation.

While cases of miRNA-mediated translation activation have been reported, it was thought to occur only under limited circumstances (e.g., in nondividing cells). This work demonstrated that miRNAs can promote translation in proliferating cells and that upregulation may be the major mode of miRNA action for some proteins. Similar studies in the future should help us better understand and utilize these fascinating noncoding RNAs.

## 3. Long Non-Coding RNAs as Indicators of Aging in Human Endothelial Cells

### Highlight by Mirolyuba Ilieva, Anja Holm and Shizuka Uchida

Aging is a natural process of changes that occur over time in living organisms, resulting in a gradual decline in physiological activities and functions. As they age, the living organisms become more prone to diseases, eventually leading to death. Thus, aging is considered as an unavoidable pathogenic process. During aging, molecular changes occur in various cell types, sometimes leading to the accumulation of undesirable molecules (e.g., reactive oxygen species) that damage cells and tissues. To uncover molecular changes associated with aging, Drekolia et al. performed RNA sequencing of endothelial cells isolated from arteries of young (average age of 20 years) and aged (average age of 80 years) individuals [2,3]. Of 4465 lncRNAs expressed in the human endothelium, 798 lncRNAs are dysregulated in advanced age. Among these differentially expressed lncRNAs, the authors focused on prostate-cancer-associated transcript 14 (PCAT14), which is localized in the nucleus of young endothelial cells, but significantly reduced in aged endothelium. By silencing PCAT14, the authors demonstrate that endothelial cell migration and sprouting capacity were reduced, without affecting the endothelial proliferative capacity. Furthermore, silencing of PCAT14 resulted in increased expression of inflammatory genes (e.g., ICAM1 and SELE) and genes relevant to endothelial cell stalk formation (e.g., JAG1 and ESM1), suggesting that PCAT14 may be important in maintaining the healthy status of the endothelium. However, the exact mechanism of action of PCAT14 is unknown. In addition, according to the latest annotation provided by the Ensembl database (https://www.ensembl.org/Homo_sapiens/Gene/Summary?db=core;g=ENSG00000280623;r=22:23536881-23547797; accessed on 16 March 2023), there are three transcripts (isoforms) of PCAT14, which needs further investigation to allow for the understanding of the function of each isoform relating this aging-related lncRNA.

## 4. Cryptic Proteins Encoded by Long Non-Coding RNAs Regulate Transcription in Cancer

### Highlight by Johanna K. DiStefano

Long non-coding RNAs are generally thought to lack protein-coding capacity, yet a growing number of studies suggests that cryptic translation within these transcripts may be important for developmental and physiological functions. In a recent issue of *Journal of Clinical Investigation*, Zheng and colleagues [4] demonstrate that lncRNA-encoded proteins may also play critical roles in the development of complex diseases.

Using a combination of ribosome profiling and CRISPR/Cas9 screening, the authors identified 28 cryptic open reading frames encoded by lncRNAs that were upregulated in human estrogen receptor-α-positive breast cancer (ER+ BC). The expression of one lncRNA, LINC00992, was associated with poor prognosis in luminal BC tumors and was therefore selected for further characterization. LINC00992 was found to encode an unannotated protein, GATA3-interacting cryptic protein (GT3-INCP), which was detected in the nucleus and cytoplasm of BC cells. The expression of LINC00992 and GTE-INCP was upregulated in ER+ cell lines and tumors. GT3-INCP promoted tumor growth in vitro and in vivo, while LINC00992 knockdown abrogated tumor formation. Of interest, GTE-INCP was shown to interact with the transcription factor GATA3, which is frequently mutated in BC and required for the estrogen-dependent proliferation of ER+ BC cells. The molecular mechanisms by which the GT3-INCP/GATA3 interaction impacts tumor formation, occur through the coregulation of protein-coding gene expression, including genes involved in estrogen response, DNA replication, and DNA repair pathways. The BC susceptibility genes, *MYB* and *PDZK1*, were identified as direct targets of GT3-INCP/GATA3 transcriptional regulation, and GT3-INCP appears to facilitate GATA3 binding to common *cis* regulatory elements.

This work presents a novel perspective of lncRNA-encoded proteins as participants in transcriptional regulation, and highlights the importance of these cryptic proteins in transcriptional regulatory networks that underlie dysregulated gene expression in cancer. The integrated genomic strategy of ER+ BC employed in this study represents a successful approach to identifying cryptic lncRNA-encoded proteins involved in other kinds of complex diseases, such as diabetes and neurodegenerative disorders.

## 5. Coding Immuno-Response from the Non-Coding Genomic Milieu

### Highlight by Agnieszka Bronisz

The long non-coding genes (lncRNAs) constitute a significant class of non-protein coding transcripts in mammalian cells. A recent discovery published in *Nature Communication* by Barczak et al. regarding the limited, but potent, lncRNA to be translated into peptides under targeted transcriptional regulation, changed the paradigm that most lncRNAs remain untranslated throughout their lifespan [5].

In this study, the authors identified transcription factor E2F1, regulating the non-coding genome upon upstream pharmacological inhibition of methyltransferase enzyme PRMT5 in cancer cells. The response of the mouse CT26 tumor microenvironment to PRMT5 inhibition consisted of concurrent infiltration of cytotoxic CD8+ T lymphocytes and an increase in the peptide antigen content of the major histocompatibility class I complex in tumor cells. Significantly, these peptides were derived from the group of lncRNAs of which the expression is monitored by E2F1. When such peptides derived from lncRNAs were administered to mice as a cancer vaccine, prior to a subcutaneous CT26 cell graft, tumor growth was delayed.

Studies have shown that small open reading frames exist in lncRNA genes with peptide encoding potential. Thus, the simultaneous deregulation of lncRNAome as a template for anti-cancer immunopeptide synthesis can be deployed as a novel anti-cancer vaccine. This study also reassesses the coding vs. non-coding potential of the genome for developing cancer immunotherapies.

## 6. A Novel LncRNA Knockout Strategy

### Highlight by Ling Yang

Knocking out a long non-coding RNA (lncRNA) presents a greater challenge compared to a protein-coding gene, as traditional methods, such as frameshift mutations, are unsuitable for lncRNAs. However, a recent study in *Nucleic Acid Research* by Zhang et al. introduced a novel lncRNA knockout strategy, BESST, by deleting the genomic DNA fragment from the branch point to the 3‘ splicing site in the last intron of the target lncRNA [6].

To validate the efficacy of BESST, eight candidate lncRNAs, comprising 2–7 exons, were selected, including HOTAIR. To knock out the target lncRNAs, a pair of small guide RNAs (sgRNAs) were designed and synthesized, flanking the branch point and 3′ splicing site in the last intron of each lncRNA. The authors found that by removing as little as 37 bp from the genome, the target lncRNA was retained within the cell nucleus and triggered a deadenylation-dependent surveillance response, leading to RNA degradation. 

Previous lncRNA knockout strategies include deletion of the promoter and first exon, removal of the whole transcript, and an insertion of polyadenylation signal. However, these strategies are often not applicable to lncRNAs with nearby or overlapping protein-coding genes. BESST presents a novel approach to conduct loss-of-function studies for lncRNAs, especially for natural antisense lncRNAs that overlap with protein-coding genes. This new technique offers a promising opportunity for future research in the lncRNA field.

## 7. An Exosomal Non-Coding RNA Encodes for a Novel Protein That Induces Chemoresistance in Colorectal Cancer

### Highlight by Yoh Asahi and Ajay Goel

Non-coding RNAs have long been recognized as genomic entities not translated into proteins. Circular RNAs (circRNAs) are also categorized as non-coding RNAs, because their structure lacks a 5′-cap and a 3′-end. However, recent advances in various research fields have begun to elucidate that a subset of circRNAs can indeed encode proteins. Within the same context, some circRNAs have recently been reported to regulate tumor biology in cancers by their ability to encode proteins. For instance, Pan and colleagues recently reported that exosomal circATG4B encodes for a novel protein (circATG4B-222aa), which results in the acquisition of chemoresistance to oxaliplatin, a key drug used for systemic therapy for colorectal cancer (CRC) [7].

Briefly, the authors focused on ATGB4 for its autophagy-inducing function in cancers associated with chemoresistance. Analyzing 15 candidate circRNAs originating from ATG4B, circATG4B was identified as the upregulated circRNA in oxaliplatin-resistant CRC tissues. High expression of circATG4B was also confirmed in chemoresistant CRC cells and exosomes secreted from such oxaliplatin-resistant cells. From a functional standpoint, circATG4B-222aa induced resistance to oxaliplatin by preventing the binding of TMED10 to circATG4B. These results were validated in additional experiments using animal models. The authors also used clinical tissue specimens from patients with CRC to suggest that the high expression of circATG4B and circATG4B-222aa was a poor predictors of recurrence after surgery and administration of oxaliplatin. 

These findings suggest the potential of circATG4B and circATG4B-222aa to serve as novel predictive biomarkers for response to oxaliplatin and potentially other treatment targets in CRC. Moreover, future studies using blood specimens from patients could provide an additional impact, considering that tumor-derived exosomal cargo is enclosed within their shell, including various genomic markers, such as circRNAs and proteins, which could be significantly superior to the cell-free fraction.

## 8. Non-Coding RNA Therapy Potentially Treats Diabetic Vasculopathy

### Highlight by Liuqing Yang

Patients with diabetes have an increased risk of limb ischemia, mostly caused by impaired angiogenesis associated with metabolic disruption under diabetic conditions. The reasons patients with diabetes are uniquely susceptible to developing peripheral vascular disease have remained elusive. Within their contribution to the *Journal of Clinical Investigation*, Tang et al. report that the lncRNA that enhances endothelial nitric oxide synthase (eNOS) expression (abbreviated as *LEENE*) associates with LEO1 and MYC, regulating angiogenic gene expression [8]. Furthermore, *LEENE* RNA effectively promotes ischemic recovery in vivo.

LncRNAs are typically expressed in a tissue-specific manner and are often dysregulated in disease. With the aim of investigating the biological significance of lncRNAs, Tang et al. determined the lncRNA expression profiles in human endothelial cells treated with proangiogenic stimuli. One lncRNA was found to be particularly highly expressed under hypoxic conditions, but suppressed under diabetic conditions, which the authors later named *LEENE*.

CRISPR/Cas9-mediated depletion of *LEENE* in mice (*LEENE*-KO mice) resulted in an impaired flow recovery and lower microvascular density, leading to reduced ischemic angiogenic responses under metabolic stress. Chromatin isolation by RNA purification (ChIRP)-seq and mass spectrometry using endothelial cells demonstrated that *LEENE* associates with LEO1, a key component of the RNA-polymerase-II-associated factor complex and MYC, a crucial transcription factor for angiogenesis. Mechanistically, *LEENE* promotes the biogenesis of proangiogenic factors by interacting with transcription factors LEO1 and MYC.

In *LEENE*-KO mice fed a high fat/high sucrose (HFHS) diet, exogenous viral delivery of human *LEENE* RNA enhanced angiogenesis and perfusion in response to ischemic injury, especially under hyperglycemia. Similarly, in endothelial cells, *LEENE* expression promotes angiogenic function and their interactions with other vascular cells, which are critical for ischemic recovery. 

These findings highlight the functional importance of *LEENE* lncRNA in vascular biology, and demonstrate the therapeutic potential of *LEENE* mimics in treating diabetic patients with vasculopathy.

## 9. An RNA Sponge to “Clean” Biofilms

### Highlight by Ashok Nuthanakanti and Alexander Serganov

In bacteria, small regulatory RNAs (sRNAs), in association with the RNA chaperone Hfq, control the expression of many genes by base-pairing with target transcripts. sRNAs participate in various processes, including competence, stress response, adaptation to nutrient availability, and antibiotic resistance and tolerance; however, global studies addressing sRNA roles in major human pathogens are lacking.

Huber et al. [9] recently employed a proximity ligation sequencing method to find Hfq-interacting sRNAs and their targets in *Vibrio cholerae* grown at low and high cell densities. This approach identified hundreds of previously unknown sRNA-target RNA interactions, demonstrating an abundance of sRNA-controlled cellular processes in this pathogen. In addition to hundreds of sRNA–mRNA interactions, the authors discovered almost one hundred of the so-called “sponge” RNAs, or sRNAs base-pairing with other sRNAs to neutralize their activity [10]. This large number of sponge RNAs correlates with the numbers obtained in the studies performed on model bacterial organisms, such as *E. coli* [11], and highlights the broad distribution of sponge-RNA-based regulation in bacteria. The authors then characterized in detail one of the sponge RNAs, termed OrrX, that base-pairs and inactivates four other sRNAs, Qrr1-4, involved in the modulation of quorum sensing. This inactivation explains the ability of *V. cholerae* to transition rapidly from low- to high-cell-density behavior, despite the relatively long half-life of the Qrr1-4 sRNAs. Thus, the study shows that sponge RNAs are crucial for collective behavior in *V. cholerae* and could have a direct impact on antibiotic resistance and tolerance, the processes dependent on the quorum sensing, and biofilm formation.

## 10. MicroRNA Expression Can Be Gender-Specific

### Highlight by Suresh K. Alahari

MicroRNAs regulate the expression of several genes that function in various biological processes. In a recent paper from the *Cell Reports* journal, Xiaojing Cui and colleagues reported that the downregulation of the expression of latexin by a microRNA determined hematopoiesis sex dimorphism [12].

Latexin regulates hematopoiesis via different mechanisms. Thrombospondin has been shown to be a new downstream target for latexin. Using a knockout animal model, the authors showed that latexin functions distinctively in male and female animals. In female mice, latexin negatively regulates hematopoietic stem cells (HSCs) through increased apoptosis and decreased self-renewal. In the case of male mice, latexin does not have an effect on apoptosis, proliferation, and regeneration of HSCs. These data indicate that latexin regulates hematopoiesis in a sex-dependent manner. To further understand the mechanism behind this gender-specific regulation, the authors used a web tool, microRNA target prediction database (miRDB), to identify the potential microRNAs that target thrombospondin, concluding that miR-93-3p was a good candidate. Next, the authors showed that miR-98-3p specifically downregulated the expression of throbospondin. Later experiments indicated that suppression of thrombospondin expression by miR-98-3p increased hematopoietic stem and progenitor cells (HSPCs) in vitro and in vivo. Finally, the authors’ data revealed that low levels of miR-98-3p in females lead to high thrombospondin expression yields and low HSPCs formation, while high expression of miR-98-3P in males leads to low levels of thrombospondin and an increased production of HPSCs.

In summary, this study reveals that sex/gender plays an important role in HSC function and hematopoiesis. In future studies, it will be crucial to show how the latexin/miR-983P/thrombospondin pathway regulates hematologic malignancies, such as leukemias, in sex dimorphism. 

## 11. LncRNAs in Non-Small Cell Lung Cancer Hallmarks

### Highlight by Chunru Lin

The lncRNA products are highly expressed in cancer, but their direct role in cancer hallmarks has been poorly characterized thus far. In an issue of *Cell Genomics*, Esposito and colleagues present a captivating story identifying the role that lncRNAs play in KRAS^+^ non-small cell lung cancer (NSCLC) [13]. Through a CRISPR-based screening pipeline for lncRNAs promoting multiple cancer hallmarks, followed by antisense oligonucleotide (ASO) screen, the authors determined that LncRNA1(*CHiLL1*) and *GCAWKR* regulate lung cancer hallmarks. *CHiLL1* and *GCAWKR* knockout by ASOs in 2D and 3D tumor models allowed for the identification of therapeutic vulnerabilities that could be exploited by potent antisense oligonucleotide inhibitors in a range of tumor models. Concomitantly, *CHiLL1* and *GCAWKR* drive distinct, but overlapping, oncogenic pathways, including MAPK, PI3K-AKT, p53, and mTORC1. The complex transcriptional networks in NSCLC were revealed to be regulated by lncRNAs, promoting cancer hallmarks (proliferation, chemoresistance, and invasion). Consequently, an ASO cocktail targeting *CHiLL1* and *GCAWKR* yielded greater-than-additive effects on NSCLC growth. 

NSCLC is a leading cause of cancer deaths due to undruggable mutations, toxicity, and therapy resistance. A long-contended topic in lncRNA-based cancer RNA therapies is the poor validation rate of preclinical lncRNA targets discovered. Of note, CRISPR lncRNA landscape screening and ASO phenotypic validation can accelerate the discovery of lncRNA therapeutic targets, as Esposito et al. compellingly illustrate. 

Taken together, the many impactful discoveries highlighted herein provide momentous evidence to advance the field of oncogenic lncRNA research. Targeting lncRNAs could truly help improve the efficacy of multiple innovative therapies currently being trialed as candidate approaches for a universal cancer cure. The benefits of the newly identified effects of *CHiLL1* and *GCAWKR* on cancer hallmarks may consequently invigorate the personalized cancer treatment, making these novel findings of great importance.

## 12. piRNAs in Extracellular Vesicles Fuel the Premetastatic Microenvironment

### Highlight by Barbara Pardini and Alessio Naccarati

The development of specific signals in the premetastatic microenvironment (PMM) has been shown to have a crucial role in promoting metastasis in certain cancers. Before arriving at the site, tumor cells can induce the “preparation” of PMM in distant locations via the release of tumor-secreted factors and tumor-shed extracellular vesicles (EVs) containing specific molecules.

The fibroblast-to-myofibroblast transition (FMT) is a phenomenon involved in vascular injury and tissue remodeling, but it is also known to be induced by tumor cells in omental fibroblasts to enhance cancer aggressiveness. The mechanism is triggered by several growth factors, cytokines, and chemokines, with TGF-β signaling playing a major role.

In a recent work, Li and colleagues reported that ovarian cancer cells, especially those with high metastatic properties, promote PMM formation by releasing EVs carrying several piwi-interacting RNAs (piRNAs) [14]. In particular, the tumor cell-secreted piR-25783 opens the way to tumor metastasis activating the TGF-β/SMAD2/SMAD3 signaling pathway in fibroblasts, which promotes the transition of these cells to myofibroblasts, along with cytokine secretion and the activation of proliferative, migratory, and invasive mechanisms that contribute to PMM.

Various studies have shown the importance of tumor-secreted EVs and their cargo as mediators in the communication between tumor and distant organs, but this represents one of the first to report the involvement of piRNAs in this function.

piR-25783, highly expressed in ovarian cancer and associated with shorter survival and prognosis, seems preferentially loaded in EVs by ovarian cancer cells. In tumor-derived EVs, this piRNA plays a role in the formation of PMM and development of metastasis in vivo and in vitro, opening a new way in the field of potential therapeutical target for ovarian carcinoma with metastasis.

## 13. LncRNA MALR Drives Liquid–Liquid Phase Separation to Promote Esophageal Cancer Process

### Highlight by Jing Jin, Beshoy Armanios and Xiao-bo Zhong

Liquid–liquid phase separation (LLPS) is a relatively new concept that explains spatiotemporal regulation in cells. Living cells assemble nucleic acids and proteins into micron-scale, liquid-like, non-membrane compartments, known as biomolecular condensates, to participate in intracellular biological activities, including chromatin remodeling, genomic stability, transcription, etc. Recent studies have suggested that liquid–liquid phase is involved in the spatiotemporal coordination of cancer cells. Long non-coding RNAs (lncRNAs) have emerged as crucial players that influence several cancer processes. However, the precise roles and mechanisms of lncRNAs in LLPS-mediated cancer progression remain largely unknown.

Jia Liu and colleagues [15] recently illustrated a complex regulation axis of macrophage-associated lncRNA (MALR)/interleukin enhancer-binding factor 3 (ILF3)-mediated LLPS that promotes the esophageal cancer process. They identified the novel lncRNA, MALR, which is highly expressed in tumor-associated macrophages (TAM) co-cultured esophageal squamous cell carcinoma (ESCC) cells and ESCC tissues. MALR showed a strong correlation with hypoxia-inducible factor 1α (HIF1α) activation and highlighted its possible role in esophageal cancer. Mechanistically, MALR directly bound to ILF3 to avoid ILF3 degradation and promoted LLPS in the nucleus by the MALR/ILF3 complex. The results suggested that LLPS is required for maintaining HIF1α mRNA stability by preventing deadenylase PARN degradation, which then promotes ESCC development. 

The interaction between a tumor and its microenvironment influences its occurrence and ability to become malignant. This study demonstrated that lncRNA is an important element to trigger LLPS in tumorigenesis through its function as a scaffold and interacting with other molecules, such as nucleic acids and proteins, to affect the cancer process in promoting mRNA stability, aerobic glycolysis, and angiogenesis in cancer cells. The emerging field of lncRNA and LLPS is providing new insights into the complex and dynamic regulation in modulating cellular processes.

## 14. Tightening the Noose: lncRNAs Regulate Chromatin Loop Formation to Promote miRNA-Driven Malignant Transformation in Glioma

### Highlight by Nikolaos Sideris, Salih Bayraktar and Leandro Castellano

The article by Evgeny Deforzh et al. [16] in *Molecular Cell*, titled “Promoter and enhancer RNAs regulate chromatin reorganization and activation of miR-10b/HOXD locus, and neoplastic transformation in glioma”, describes how the coordinated activity of two lncRNAs controls 3D chromatin reorganization to activate the HOXD/miR-10b cluster in glioma and drive malignant transformation. MiR-10b is an oncogenic microRNA (miRNA) highly expressed in glioma, critical for cancer cell survival and proliferation, and located within the HOXD cluster. Under normal conditions, the entire HOXD/miR-10b locus is epigenetically repressed in developed neural cells. In this article, researchers identify the promoter-transcribed HOXD-AS2 and distal enhancer-transcribed LINC01116 as the driving forces for mir-10b expression in glioma. Both lncRNAs have been previously associated with microRNA sponging and disease progression in various cancers, including glioma. Interestingly, by combining 4C-seq and single-molecule FISH (smFISH), the authors demonstrate that the co-localization in the nuclei and interaction between these lncRNAs orchestrates locus reactivation through CTCF-cohesin mediated chromatin looping, via recruitment of CTCF and RAD21. Knockdown of either lncRNA or independent CRISPR editing of the CTCF binding sites was shown to abrogate this effect. Conversely, CRISPR activation of the eRNA alone in normal human astrocytes induced topological changes and expression of miR-10b, among other genes, promoting a similar phenotype in glioma. This study delves into the largely unexplored area of chromatin reorganization in cancer and provides a novel mechanism for the development of glioma, a highly heterogeneous cancer. These findings pave the way for new therapeutic avenues for glioma patients.

## 15. Translation of Telomeric RNA (TERRA) Can Produce Valine–Arginine and Glycine–Leucine Dipeptide Repeat Proteins

### Highlight by André P. Gerber

It has been known for a decade that eukaryotic telomers are transcribed and produce a G-rich structural RNA, termed TERRA, that does not code for proteins. A recent study by Al-Turki and Griffith [17] has now challenged this view by considering that disease-associated nucleotide repeat expansions in RNA could be translated to generate homopeptide or dipeptide repeat proteins in a process called Repeat Associated Non-ATG (RAN) translation. Consequently, the authors wondered whether TERRA could also be translated and produce highly charged VR and hydrophobic GL dipeptides.

Initially, they tested the biochemical properties of synthetic VR and GL micro-peptides, and found that they can assemble into long filaments with amyloid properties and VR peptides binding to RNA and ssDNA. An antibody specifically recognizing VR dipeptides was produced to characterize the aggregation properties and localization in different cell lines using high-resolution confocal microscopy. VR signals appeared as punctate spots, mainly in the nucleus, and were increased in cells with higher TERRA levels. A reduction of TERRA in cells through locked nucleic acid (LNA) GAPmeRs led to the redistribution of VR peptides into larger nuclear foci; while knock-down of TRF2—a protein protecting telomer ends and known to increase cytoplasmic TERRA levels—led to significant increase in VR peptides in the cytoplasm. Hence, the distribution and abundance of VR dipeptides seem to depend on TERRA levels.

Overall, the data suggest that TERRA could be translated into previously overlooked dipeptides that may alter nucleic acid metabolisms and general protein synthesis and trigger inflammatory response. The authors further speculate that VR and GL dipeptide proteins could act as signals for dysfunctional telomeres. Thus, further studies on the biological activity of these micropeptides could lead to new fundamental insights into the biology of normal and transformed cells. 

## 16. Good Folks Again: A Circular RNA Plays a Protective Role in Diabetes and Obesity

### Highlight by He Lin and Simon J. Conn

Type II diabetes mellitus (T2DM) is one of the most common diseases worldwide, with the prevalence predicted to reach 7% globally by 2030 with increasing cases of clinical obesity. In a recent article published in *Nature Communications*, Liu et al. reported that a circular RNA, *circGlis3*, plays a protective role in T2DM pathology by promoting insulin secretion and decreasing obesity-associated β-cell apoptosis [18].

In this study, *CircGlis3* was found to be increased in mouse models of obesity and diabetes, and its orthologue to be more abundant in the serum of human patients with impaired glucose tolerance and T2DM. *CircGlis3* was found to interact with the pro-apoptotic protein SCOTIN to reduce apoptosis in the mouse insulinoma cell line, MIN6. It was also found to increase insulin secretion and improve glucose tolerance in vivo, by increasing the expression of *NeuroD1* and *Creb1* through a sponging microRNA, miR-124-3p. The biogenesis of *circGlis3* is regulated by Quaking, which is known to drive approximately one-third of all circRNAs [19]. However, mature *circGlis3* also interacts with another known circRNA biogenesis protein, FUS, which the authors reveal to sequester *circGlis3* into stress granules, thereby mitigating its protective role.

While there are a number of reports of circRNAs as potential biomarkers, this study is unique in that a single circRNA has also been shown to be an intracellular protectant for pancreatic β-cell dysfunction. While the authors identify some limitations of their study, further research should help us better comprehend the potential biomarker and therapeutic applications of this and other circular RNA molecules in a variety of human diseases.

## 17. BSG/EMMPRIN/CD147 Marks a Distinct Class of miRNA-Enriched Extracellular Vesicles and Is a Promising Cancer Biomarker

### Highlight by Doha Magdy Mostafa Sleem and Lisa Timmons

Extracellular Vesicles (EVs), once regarded as cellular debris, now encompass a diverse population of membrane-bound particles. These circulating particles can be categorized based on size (ranging from 20 nm to 20 μm) and their cargo content, which can include membrane proteins, lipids, and specific miRNA sequences. Furthermore, EVs are characterized by their cellular and endomembrane compartment origins, as well as their biogenesis (whether they are directly secreted from the plasma membrane or formed through the multi-vesicular body pathway, for example). In 2020, a study involving hundreds of matched normal and cancerous human tissues and serum samples identified several EV protein signatures strongly correlated with cancer, including BSG/EMMPRIN/CD147 [20].

Recently, Ko et al. [21] conducted elegant experiments supporting the use of CD147-containing EVs as a cancer biomarker, particularly as their numbers increase with respect to disease progression. The study found that the CD147 EVs are distinct from the more familiar tetraspanin EVs, and likely originate from the plasma membrane as microvesicles, and not multivesicular body exosomes, which is in-keeping with their larger size. CD147 EVs from cultured cells contain significantly more miRNAs than tetraspanin EVs (8–17 fold more), and the RNA-binding protein, hnRNP A2/B1, contributes to this difference. The study further confirmed that CD147 EVs can transmit information, as EVs isolated from miR-302-overexpressing donor cells could silence an engineered reporter in recipient cells. Importantly, the expression levels of miRNA populations in CD147 EVs isolated from patient body fluids matched those of patient tumors.

A significant objective of the field is to identify extracellular vesicle subpopulations that can serve as disease biomarkers. Another crucial goal is to efficiently extract extracellular vesicles from patient body fluids for a thorough examination, to provide insights into disease development and progression. Utilizing a simple immunocapture technique, this study successfully identified CD147 EVs as a particular extracellular vesicle population that becomes increasingly more abundant as cancer progresses and is enriched in miRNAs, which is a notable advancement towards achieving these goals.
